# Clinical characteristics and predictive value of lower CD4^+^T cell level in patients with moderate and severe COVID-19: a multicenter retrospective study

**DOI:** 10.1186/s12879-020-05741-w

**Published:** 2021-01-12

**Authors:** Xue-song Wen, Dan Jiang, Lei Gao, Jian-zhong Zhou, Jun Xiao, Xiao-cheng Cheng, Bin He, Yue Chen, Peng Lei, Xiao-wei Tan, Shu Qin, Dong-ying Zhang

**Affiliations:** 1 Department of Cardiovascular Medicine, The First Affiliated Hospital of Chongqing Medical University, No 1, Youyi Road, Chongqing, 400016 China; 2 Department of Cardiovascular Medicine, Chongqing University Center Hospital, Chongqing, 400014 China

**Keywords:** CD4^+^T cells, COVID-19, In-hospital death, SARS-CoV-2

## Abstract

**Background:**

In December 2019, coronavirus disease 2019 (COVID-19) caused by severe acute respiratory syndrome coronavirus 2 (SARS-CoV-2) emerged in Wuhan, Hubei, China. Moreover, it has become a global pandemic. This is of great value in describing the clinical symptoms of COVID-19 patients in detail and looking for markers which are significant to predict the prognosis of COVID-19 patients.

**Methods:**

In this multicenter, retrospective study, 476 patients with COVID-19 were enrolled from a consecutive series. After screening, a total of 395 patients were included in this study. All-cause death was the primary endpoint. All patients were followed up from admission till discharge or death.

**Results:**

The main symptoms observed in the study included fever on admission, cough, fatigue, and shortness of breath. The most common comorbidities were hypertension and diabetes mellitus. Patients with lower CD4^+^T cell level were older and more often male compared to those with higher CD4^+^T cell level. Reduced CD8^+^T cell level was an indicator of the severity of COVID-19. Both decreased CD4^+^T [HR:13.659; 95%CI: 3.235–57.671] and CD8^+^T [HR: 10.883; 95%CI: 3.277–36.145] cell levels were associated with in-hospital death in COVID-19 patients, but only the decrease of CD4^+^T cell level was an independent predictor of in-hospital death in COVID-19 patients.

**Conclusions:**

Reductions in lymphocytes and lymphocyte subsets were common in COVID-19 patients, especially in severe cases of COVID-19. It was the CD8^+^T cell level, not the CD4^+^T cell level, that reflected the severity of the patient’s disease. Only reduced CD4^+^T cell level was independently associated with increased in-hospital death in COVID-19 patients.

**Trial registration:**

Prognostic Factors of Patients With COVID-19, NCT04292964. Registered 03 March 2020. Retrospectively registered.

**Supplementary Information:**

The online version contains supplementary material available at 10.1186/s12879-020-05741-w.

## Background

In December 2019, an outbreak of coronavirus disease 2019 (COVID-19), an acute respiratory illness caused by severe acute respiratory syndrome coronavirus 2 (SARS-CoV-2), was detected in Chinese mainland. Although the overall case fatality rate for COVID-19 patients is relatively low [1], the number of deaths associated with COVID-19 has already exceeded the sum of SARS and MERS, which has brought extraordinary harm to human beings [2]. Moreover, the fatality rate of patients with severe COVID-19 is higher, and the harm is bound to be more critical [3]. This is of great value in describing the clinical symptoms of COVID-19 patients in detail and finding markers to predict the prognosis of COVID-19 patients.

The decrease of T-lymphocytes in peripheral blood was a normal laboratory characteristic in patients with COVID-19, especially in patients with severe COVID-19 [4, 5]. A later study recruited 21 patients with COVID-19, 11 patients with severe COVID-19 and 10 patients with moderate COVID-19. The study showed that the absolute numbers of T-lymphocytes, CD4^+^T cells, and CD8^+^T cells were decreased in almost all COVID-19 patients, with significantly lower numbers in patients with severe COVID-19 (294.0, 177.5, and 89.0× 10^6^/L) than in patients with moderate COVID-19 (640.5, 381.5, and 254.0× 10^6^/L). Meanwhile, the number of B-lymphocytes did not decrease but rather tended to increase in most patients. This phenomenon suggested that SARS-CoV-2 infection may mainly affect T-lymphocytes, especially CD4^+^T cells and CD8^+^T cells [5]. T-lymphocytes play a crucial role in antiviral immunity. The CD4^+^T lymphocyte subsets secreted a high level of effector cytokines, particularly interferon-γ (IFN-γ), which were essential for virus clearance [6, 7]. A previous study also showed that a dramatic decrease in total lymphocytes indicated that the coronavirus consumed immune cells and destructed cellular immune function [8]. However, there is insufficient evidence as to whether CD4^+^T cells can predict the prognosis in patients with COVID-19.

## Methods

### Subjects

**Fig. 1:**
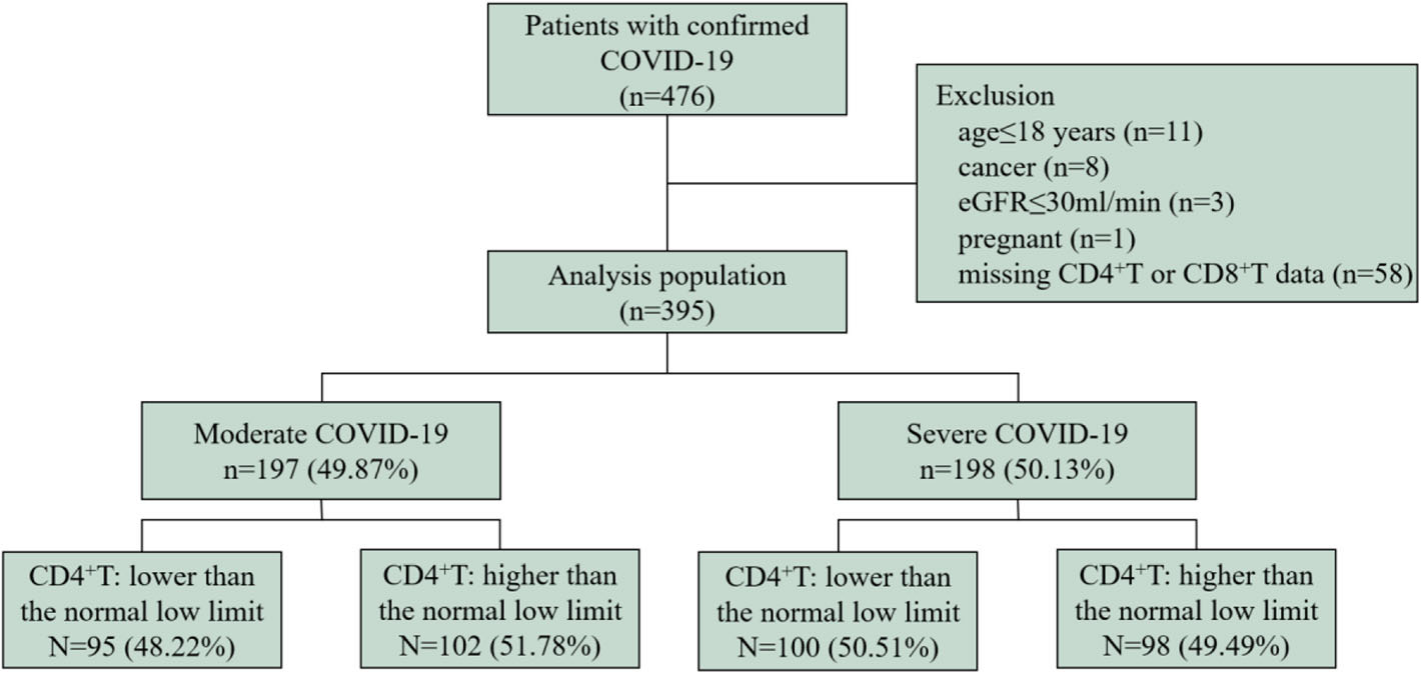
Flow diagram of Patient Recruitment

Medical records from 476 patients with confirmed COVID-19 were collected in Hubei General Hospital and Chongqing Three Gorges Central Hospital. Missing CD4^+^T cell level or CD8^+^T cell level data (*n*=58), malignant tumor (*n*=8), younger than 18 years (*n*=11), eGFR≤30 ml/min (*n*=3), and pregnant (*n*=1) were excluded, patients with immune system diseases or HIV, which may affect lymphocyte subsets, were also excluded. Finally, 395 patients with COVID-19 were analyzed in this study (Fig. 1). The positive infected patients were confirmed by testing new coronavirus nucleic acid by real-time fluorescent Polymerase Chain Reaction (RT-PCR). Patients with severe COVID-19 were defined according to the New Coronavirus Pneumonia Prevention and Control Program issued by the National Health Commission of the People’s Republic of China (5th edition). Patients with respiratory distress (respiratory rates ≥30 breaths/minute or resting oxygen saturation ≤93% or partial pressure of arterial oxygen (PaO2)/ inspired oxygen fraction (FiO2) ≤300 mmHg or respiratory failure requiring mechanical ventilation) were defined as severe COVID-19, and the remaining patients were defined as moderate COVID-19 patients. According to the levels of CD4^+^T cell, CD8^+^T cell, and lymphocyte (the lower limit of the laboratory reference value), the patients with COVID-19 were divided into lower group and higher group. The study was a multicenter, retrospective, observational registry with clinicaltrials.gov identifier NCT04292964. All study procedures were approved by the local ethics committee (approval number 20200701). All data were collected by experienced researchers using blinded methods.

### Baseline data and follow-up

Demographic and clinical characteristics were collected from the electronic medical record system. Data collection for laboratory results was defined by the results of the first test after hospital admission. The absolute number of lymphocytes was measured by an automatic blood cell analyzer. Peripheral blood lymphocyte subsets were detected by flow cytometry (BD FACSCanto II flow cytometer, BD Biosciences, USA). Data from both clinical centers were standardized, and the standardized tables were used to collect clinical data from COVID-19 patients. All COVID-19 patients in the study were followed-up from admission to death or discharge from the hospital. The outcome was defined as in-hospital death.

### Statistical analysis

Continuous data were expressed as mean ± standard deviation (SD) or median (interquartile range) according to the distribution. Categorical variables were expressed as frequency rates with percentages. Continuous variables were compared using the independent samples T-test (normal distribution) and the Mann-Whitney U-test (nonnormal distribution). Categorical data were tested using the Chi-square test and Fisher’s exact Chi-square test. Univariate analyses and multivariate analyses were performed using Cox proportional-hazards models to determine the relationship between CD4^+^T cell level and in-hospital death. Kaplan-Meier survival analysis with a log-rank test was performed to estimate the cumulative survival rate of groups with higher or lower CD4^+^T cell level. Statistical analyses were performed by the IBM SPSS Statistics 26.0 software (SPSS, Chicago, IL, USA). P (two-tailed) value less than 0.05 was considered statistical significance.

## Results

### Baseline characteristics

Baseline characteristics are shown in Table 1. The mean age of all COVID-19 patients was 53 years, and 204 patients (51.6%) were male. In these patients, fever on admission (263, 66.6%) was the most common symptom. Cough, shortness of breath, fatigue, and sputum production were present in 257 patients (65.1%), 118 patients (29.9%), 107 patients (27.2%), and 102 patients (25.9%), respectively. In our study, Headache (36, 9.1%), nausea or vomiting (36, 9.1%), myalgia or arthralgia (34, 8.6%), sore throat (22, 5.6%), and chill (7, 6.7%) were rare. The most common comorbidities were hypertension (102, 25.8%) and diabetes mellitus (47, 11.3%). The rates of coronary heart disease, hepatitis B infection, and chronic obstructive pulmonary disease were 6.4% (25/392), 2.3% (9/392), and 1.5% (6/392), respectively.

**Table 1:** Baseline characteristics of different degrees of CD4^+^T cell level in all COVID-19 patients

Variables	All (*N*=395)	CD4+T: lower group (*N*=195)	CD4+T: higher group (*N*=200)	*P*	Missing data
Baseline					
Male/female(n)	204/191	115/80	89/111	0.004	
Age (years)	53.1±15.7	55.0±16.5	51.3±14.8	0.033	
Temperature (°C)	36.8 (36.5–37.3)	36.9 (36.6–37.6)	36.8 (36.5–37.1)	0.036	31 (7.8%)
Heart rate (min)	85.0 (77.0–94.0)	85.0 (78.0–96.0)	84.5 (76.0–92.0)	0.103	4 (1.0%)
SBP (mmHg)	126.0 (116.0–136.0)	126.0 (115.0–136.5)	126.0 (117.0–136.0)	0.577	6 (1.5%)
DBP (mmHg)	78.0 (70.0–85.0)	76.0 (70.0–85.0)	78.0 (71.0–85.0)	0.741	6 (1.5%)
Symptoms and signs---No, %					
Fever on admission	263 (66.6%)	141 (72.3%)	122 (61.0%)	0.017	
Nasal congestion	2 (0.5%)	2 (1.0%)	0 (0%)	0.243	
Headache	36 (9.1%)	20 (10.3%)	16 (8.0%)	0.436	
Cough	257 (65.1%)	138 (70.8%)	119 (59.5%)	0.019	
Sore throat	22 (5.6%)	10 (5.1%)	12 (6.0%)	0.706	
Sputum production	102 (25.9%)	56 (28.9%)	46 (23.0%)	0.184	1 (0.3%)
Fatigue	107 (27.2%)	59 (30.4%)	48 (24.0%)	0.153	1 (0.3%)
Shortness of breath	118 (29.9%)	75 (38.5%)	43 (21.5%)	< 0.001	
Nausea or vomiting	36 (9.1%)	23 (11.8%)	13 (6.5%)	0.068	
Myalgia or arthralgia	34 (8.6%)	20 (10.3%)	14 (7.0%)	0.249	
Chill	12 (3.0%)	8 (4.1%)	4 (2.0%)	0.223	
Throat congestion	3 (0.8%)	0 (0%)	3 (1.5%)	0.248	
Coexisting disorders---No, %					
Diabetes	47 (11.9%)	22 (11.3%)	25 (12.5%)	0.709	
Hypertension	102 (25.8%)	48 (24.6%)	54 (27.0%)	0.588	
Coronary heart disease	25 (6.4%)	15 (7.7%)	10 (5.1%)	0.277	3 (0.8%)
Hepatitis B infection	9 (2.3%)	6 (3.1%)	3 (1.5%)	0.334	3 (0.8%)
COPD	6 (1.5%)	5 (2.6%)	1 (0.5%)	0.119	3 (0.8%)
Laboratory findings					
WBC (×10^9^/L)	5.3 (4.2–7.0)	5.0 (3.8–7.0)	5.6 (4.5–7.0)	0.008	2 (0.5%)
Hb (g/L)	131.0 (118.5–143.0)	132.0 (117.0–143.0)	129.0 (120.0–142.3)	0.809	2 (0.5%)
PLT (× 10^9^/L)	189.0 (145.5–252.0)	160.0 (129.0–214.0)	220.5 (170.0–364.0)	< 0.001	2 (0.5%)
LYM (×10^9^/L)	1.1 (0.8–1.5)	0.8 (0.6–1.0)	1.5 (1.2–1.8)	< 0.001	6 (1.5%)
LYM< 1.1×10^9^/L	199 (51.2%)	163 (84.5%)	27 (13.8%)	< 0.001	6 (1.5%)
ALT (U/L)	23.0 (15.0–39.0)	24.1 (15.4–38.8)	22.0 (15.0–39.0)	0.388	4 (1.0%)
Cr (umol/L)	64.0 (53.0–78.0)	66.5 (56.0–79.0)	61.0 (50.0–77.0)	0.005	5 (1.3%)
D-dimer (mg/L)	0.43 (0.24–0.99)	0.50 (0.28–1.12)	0.38 (0.22–0.84)	0.023	14 (3.5%)
K (mmol/L)	4.0 (3.7–4.3)	4.0 (3.6–4.3)	4.1 (3.7–4.3)	0.243	6 (1.5%)
Hs-CRP (mg/L)	5.0 (2.2–22.9)	8.2 (5.0–48.5)	4.9 (1.1–7.0)	< 0.001	45 (11.4%)
PCT (ng/ml)	0.05 (0.03–0.08)	0.06 (0.04–0.11)	0.04 (0.02–0.06)	< 0.001	21 (5.3%)
CD4^+^T cell level	410.0 (265.0–567.0)	262.0 (188.0–325.0)	564.0 (478.5–716.0)	< 0.001	
CD8^+^T cell level	246.0 (154.0–348.0)	168.0 (107.0–250.0)	322.0 (244.3–443.5)	< 0.001	
CD4/CD8 ratio	1.6 (1.2–2.2)	1.4 (1.1–1.9)	1.8 (1.4–2.3)	< 0.001	
Abnormalities on chest CT---No, %					
Ground-glass opacity	170 (48.7%)	78 (46.7%)	92 (50.5%)	0.473	46 (11.6%)
Local patchy shadowing	135 (38.7%)	71 (42.5%)	64 (35.2%)	0.159	46 (11.6%)
Treatment---No, %					
Oxygen inhalation	328 (84.3%)	174 (90.2%)	154 (78.6%)	0.002	6 (1.5%)
Glucocorticoids	94 (23.8%)	64 (32.8%)	30 (15.0%)	< 0.001	
Antiviral treatment	388 (98.2%)	191 (97.9%)	197 (98.5%)	0.721	
Intravenous immunoglobulin	71 (18.2%)	37 (19.2%)	34 (17.3%)	0.625	5 (1.3%)
Antibiotic treatment	179 (45.3%)	112 (57.4%)	67 (33.5%)	< 0.001	
Antifungal treatment	4 (1.0%)	2 (1.0%)	2 (1.0%)	1.000	
Clinical outcome					
Death (No, %)	27 (6.8%)	25 (12.8%)	2 (1.0%)	< 0.001	

*Abbreviations*: *SBP* systolic blood pressure, *DBP* diastolic blood pressure, *COPD* Chronic obstructive pulmonary disease, *WBC* white blood cell, *Hb* Hemoglobin, *PLT* platelet, *LYM* lymphocyte, *ALT* alanine aminotransferase, *Cr* creatinine, *Hs-CRP* hypersensitive C-reactive protein, *PCT* procalcitonin

The 395 COVID-19 patients were divided into two groups according to the lower limit of laboratory CD4^+^T cell level reference value: the lower CD4^+^T cell level group and the higher CD4^+^T cell level group. Patients in the lower group were older (55.0±16.5 vs 51.3±14.8, *P*=0.033), more male (115/195 [59.0%] vs 89/111 [44.5%], *P*=0.004), and more likely to have shortness of breath (75/195 [38.5%] vs 43/200 [21.5%], *P*< 0.001) and fever on admission (141/195 [72.3%] vs 122/200 [61.0%], *P=*0.017). There was no apparent difference between the two groups in the prevalence of comorbidities such as hypertension, diabetes mellitus, coronary heart disease, hepatitis B infection, and chronic obstructive pulmonary disease. The same trend were observed in the analysis of patients with moderate and severe COVID-19 alone (Supplementary Table 1, Supplementary Table 2).

### Laboratory and radiographic findings

In these 395 COVID-19 patients, Hs-CRP (5.0 [2.2–22.9] mg/L) and PCT (0.05 [0.03–0.08] ng/ml) levels were elevated, while lymphocytes, CD4^+^T and CD8^+^T cell levels were all within the standard ranges (Table 1). In patients with moderate COVID-19, only Hs-CRP level was elevated. (Supplementary Table 1). In patients with severe COVID-19, the levels of Hs-CRP, PCT, and D-dimer were elevated, whereas the levels of lymphocytes, CD4^+^T cell, and CD8^+^T cell were decreased (Supplementary Table 2). According to lung CT (computed tomography, CT) findings, the proportions of ground-glass opacity and patchy local shadowing in all COVID-19 patients were 48.7% (170/349) and 38.7% (135/349), respectively.

**Fig. 2:**
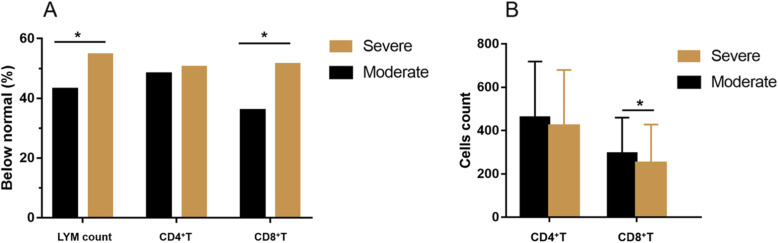
**a**: The histogram shows the proportion of moderate and severe COVID-19 patients with lymphocyte, CD4^+^T and CD8^+^T cell levels below the lower limit of normal count; **b**: The histogram shows the number of CD4^+^T cells and CD8^+^T cells in moderate and severe COVID-19 patients

In terms of laboratory findings, compared with patients in the group with higher CD4^+^T cell level, patients in the group with lower CD4^+^T cell level had lower lymphocytes levels (0.8 [0.6–1.0] vs. 1.5 [1.2–1.8], *P*< 0.001, cells× 10^9^/L), CD8^+^T cell level (168.0 [107.0–250.0] vs. 322.0 [244.3–443.5], *P*< 0.001, cells/ul) and CD4/CD8 ratio (1.4 [1.1–1.9] vs. 1.8 [1.4–2.3], *P*< 0.001), but higher Hs-CRP level (8.2 [5.0–48.5] vs. 4.9 [1.1–7.0], *P*< 0.001, mg/L) and PCT level (0.06 [0.04–0.11 vs. 0.04 [0.02–0.06], *P*< 0.001, ng/ml) (Table 1). Analysis of patients with moderate and severe COVID-19 alone, there was no significant change in the proportion of decreased CD4^+^T cell level. However, the proportion of decreased CD8^+^T cell level was significantly higher in patients with severe COVID-19 than in patients with moderate COVID-19 (51.5% [102/198] vs. 36.0% [71/197]) (Fig. 2a). It was also found that CD8^+^T cell level was more commonly reduced in patients with severe COVID-19 than in patients with moderate COVID-19 (Fig. 2b).

In terms of computed tomography findings, the moderate COVID-19 patients in the lower CD4^+^T cell level group more often represented as patchy local shadowing (45 [47.4%] vs. 33 [32.4%], *P*=0.031) compared with the moderate COVID-19 patients in the higher CD4^+^T cell level group. Ground-glass opacity and local patchy shadowing did not differ between the two groups in the whole patient population (Table 1).

### Treatment and clinical outcome

In all cases, the use of oxygen inhalation and mechanical ventilation were 84.3% (328/389) and 7.7% (30/388), respectively. The most common treatment was antiviral therapy (388/395, 98.2%), followed by antibiotic therapy (179/395, 45.3%), glucocorticoids therapy (94/395, 23.8%), intravenous immunoglobulin therapy (71/395, 18.2%), and only four patients (4/395, 1.0%) were treated with antifungal drugs. During the follow-up period, 27 patients died (27/395, 6.8%) and the rest were discharged (368/395, 93.2%).

Patients in the lower group required more oxygen inhalation (90.2% [174/193] vs. 78.6% [154/196], *P*=0.002), mechanical ventilation (13.5% [26/193] vs. 2.1% [4/195], *P*< 0.001), antibiotic therapy (57.4% [112/195] vs. 33.5% [67/200], *P*< 0.001), and glucocorticoid therapy (32.8% [64/195] vs. 15.0% [30/200], *P*< 0.001). Other treatments were similar in both groups, such as antiviral therapy, intravenous immunoglobulin therapy, and antifungal therapy. The case in-hospital death rate was significantly higher in patients with lower CD4^+^T cell level than in those with higher CD4^+^T cell level (12.8% [25/195] vs. 1.0% [2/200], *P*< 0.001). Details of the treatment of patients with moderate and severe COVID-19 were shown in Supplementary Table 1 and Supplementary Table 2.

### Survival curves of in-hospital death

**Fig. 3:**
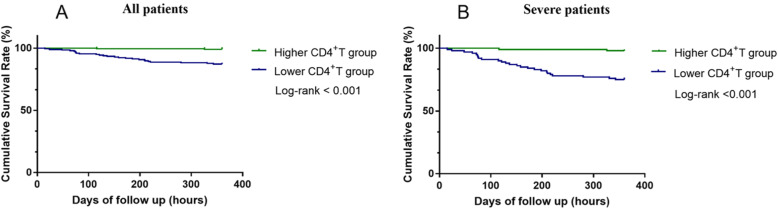
Kaplan-Meier plots shows the survival rate of COVID-19 patients who were stratified into two groups according to CD4^+^T cell level. (green line, higher CD4^+^T cell level group; blue line, lower CD4^+^T cell level group)

Kaplan-Meier survival curves for COVID-19 patients grouped by CD4^+^T cell level are shown in Fig. 3. During follow-up, the in-hospital death rate was higher in the group with lower CD4^+^T cell level than in the group with higher CD4^+^T cell level (log rank< 0.001). The same trend was also found in patients with severe COVID-19 (log rank< 0.001). Kaplan-Meier survival analysis was not performed for patients with moderate COVID-19 as there were no moderate COVID-19 patients who died during follow-up.

### Results of Cox proportional hazards analyses of in-hospital death

Cox regression analysis was performed to test the relationship between the variables and in-hospital death in patients with COVID-19 (Supplementary Table 3). Univariate analysis revealed a 13.659-fold increased risk of in-hospital death in patients with lower CD4^+^T cell level compared to those with higher CD4^+^T cell level (hazard ratio (HR):13.659; 95% confidence intervals (CI):3.235–57.671). Also, age, history of hypertension and COPD, white blood cell and lymphocyte levels, and lower CD8^+^T cell level (HR: 10.883; 95%CI: 3.277–36.145) were associated with an increased risk of in-hospital death in patients with COVID-19 (Supplementary Table 3).

**Fig. 4:**
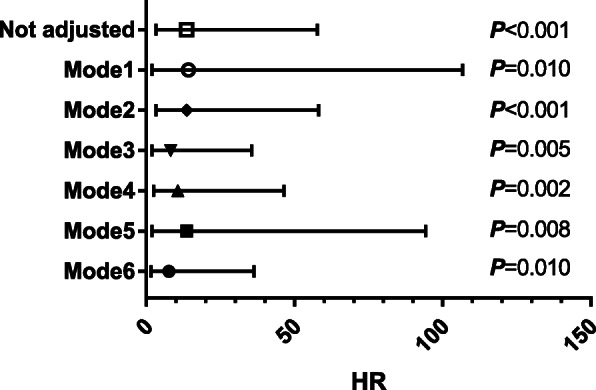
Forest plots of multivariate Cox proportional-hazards regression analyze the effect of baseline variables on in-hospital death in all patients with COVID-19. Mode1: adjusted sex, age, and temperature; Mode2: adjusted hypertension, diabetes, and shortness of breath; Mode3: adjusted white blood cell, platelet, and Creatinine; Mode4: adjusted hypersensitive C-reactive protein, procalcitonin, and D-dimer; Mode5: adjusted the group with lower CD8^+^T cell level, CD4/CD8 ratio, and the group with lower lymphocyte level; Mode6: adjusted age, hypertension, shortness of breath, white blood cell, platelet, D-dimer, and CD4/CD8 ratio

Multivariate Cox proportional hazards regression survival analysis was performed to identify the independent factors associated with prognosis (Table 2). After adjusting for age, sex, and temperature (Mode 1), the HR for in-hospital death in the group with lower CD4^+^T cell level was 14.182 (95%CI: 1.884–106.786, *P*=0.010). After adjusting for history of hypertension and diabetes, shortness of breath (Mode 2), the HR for in-hospital death in the group with lower CD4^+^T cell level was 13.631 (95%CI: 3.190–58.243, *P*< 0.001). After adjusting for white blood cells, platelets, and creatinine (Mode 3), the HR for in-hospital death in the group with lower CD4^+^T cell level was 8.170 (95%CI: 1.877–35.566, *P*=0.005). After adjusting for Hs-CRP, PCT, and D-dimer (Mode4), the HR for in-hospital death in the group with lower CD4^+^T cell level was 10.644 (95%CI: 2.439–46.458, *P*=0.002). After adjusting for CD8^+^T cells and lymphocytes (Mode 5), the HR for in-hospital death in the group with lower CD4^+^T cell level was 13.650 (95%CI: 1.976–94.279, *P*=0.008); Furthermore, in this model, the HR for in-hospital death in the group with lower CD8^+^T cell level was 2.873 (95%CI: 0.771–10.709, *P*=0.116) after adjusting for other factors, we thus concluded that reduced CD4^+^T cell level was a better predictor of in-hospital death. After adjusting for age, history of hypertension, shortness of breath, white blood cells, platelets, D-dimer, and CD4/CD8 ratio (Mode 6), the HR for in-hospital death in the group with lower CD4^+^T cell level was 7.656 (95%CI: 1.610–36.396, *P*=0.010). Multivariate analysis showed that a decreased level of CD4^+^T cell was an independent risk factor for in-hospital death. Variables like age, white blood cell, and shortness of breath also showed significance for independently predicting in-hospital death in this study (Fig. 4). Similarly, Cox proportional hazards analysis was performed in patients with severe COVID-19, and the results also suggested that decreased CD4^+^T cell level was an independent risk factor for in-hospital death (Supplementary Table 4, Supplementary Table 5, Supplementary Figure 1).

**Table 2:** Results of multivariate Cox proportional-hazards regression analyzing the effect of baseline variables on in-hospital death in all patients with COVID-19

Mode	HR (95%CI)	*P*
Not adjusted CD4^+^T level, lower vs. higher	13.659 (3.235–57.671)	< 0.001
Mode 1		
CD4^+^T cell level, lower vs. higher	14.182 (1.884–106.786)	0.010
Sex, male vs. female	1.383 (0.561–3.406)	0.481
Age, per 1 year	1.093 (1.052–1.135)	< 0.001
Temperature, per 1 °C	0.777 (0.445–1.354)	0.372
Mode 2		
CD4^+^T cell level, lower vs. higher	13.631 (3.190–58.243)	< 0.001
Hypertension, yes vs. no	5.823 (2.595–13.070)	< 0.001
Diabetes, yes vs. no	0.824 (0.322–2.113)	0.688
Shortness of breath, yes vs. no	7.848 (2.942–20.934)	< 0.001
Mode 3		
CD4^+^T cell level, lower vs. higher	8.170 (1.877–35.566)	0.005
WBC, per 1×10^9^/L	1.294 (1.193–1.404)	< 0.001
PLT, per 1×10^9^/L	0.992 (0.987–0.997)	0.003
Cr, per 1 umol/L	1.002 (0.995–1.009)	0.576
Mode 4		
CD4^+^T cell level, lower vs. higher	10.644 (2.439–46.458)	0.002
Hs-CRP, per 1 mg/L	0.989 (0.974–1.005)	0.193
PCT, per 1 ng/ml	1.017 (0.925–1.118)	0.724
D-dimer, per 1 mg/L	1.028 (1.018–1.038)	< 0.001
Mode 5		
CD4^+^T cell level, lower vs. higher	13.650 (1.976–94.279)	0.008
CD8^+^T cell level, lower vs. higher	3.159 (0.853–11.707)	0.085
CD4/CD8 ratio, per 1 unit	1.422 (1.105–1.830)	0.006
LYM level, lower vs. higher	0.996 (0.306–3.243)	0.994
Mode 6		
CD4^+^T cell level, lower vs. higher	7.656 (1.610–36.396)	0.010
Age, per 1 year	1.074 (1.034–1.115)	< 0.001
Hypertension, yes vs. no	2.031 (0.766–5.386)	0.154
Shortness of breath, yes vs. no	3.435 (1.167–10.114)	0.025
WBC, per 1×10^9^/L	1.224 (1.097–1.366)	< 0.001
PLT, per 1× 10^9^/L	0.996 (0.991–1.001)	0.149
D-dimer, per 1 mg/L	0.997 (0.992–1.002)	0.207
CD4/CD8 ratio, per 1 unit	1.106 (0.793–1.542)	0.552

*Abbreviations*: *WBC* white blood cell, *PLT* platelet, *Cr* creatinine, *Hs-CRP* hypersensitive C-reactive protein, *PCT* procalcitonin, *LYM* lymphocyte

## Discussion

This study revealed the relationship between lymphocyte subpopulations and the severity of COVID-19 or in-hospital mortality in patients with COVID-19. The main symptoms observed in the study included fever on admission, cough, fatigue, and shortness of breath. The most common comorbidities were hypertension and diabetes mellitus. Patients with lower CD4^+^T cell level were older and more often male than those with higher CD4^+^T cell level. Lymphocyte levels and CD8^+^T cell level were significantly lower in the lower CD4^+^T cell level group. Reduced CD8^+^T cell level was an indicator of the severity of COVID-19. Reduced CD4^+^T and CD8^+^T cell levels were both associated with in-hospital death, but only decreased CD4^+^T cell level was an independent predictor for in-hospital death in COVID-19 patients.

We found the reduction of CD8^+^T cell level was associated with the severity of COVID-19. Previous studies suggested that CD4^+^T and CD8^+^T cell levels were reduced in the vast majority of patients with severe or moderate COVID-19 [5, 9, 10]. Reduced levels of CD4^+^T and CD8^+^T cell were not only associated with the severity of COVID-19, but also with poor outcomes [9, 11]. In this study, we found that decreased CD4^+^T and CD8^+^T cell levels were common in patients with COVID-19; there was no significant difference in decreased CD4^+^T cell level between patients with moderate and severe COVID-19, while patients with severe COVID-19 were more likely to have decreased CD8^+^T cell level, suggesting that decreased CD8^+^T cell level may reflect the severity of the disease. This result was similar to a previous report which pointed out that the decrease of CD8^+^T lymphocyte subsets level was associated with the severity of COVID-19 [12]. The reason may be that CD8^+^T cytotoxic cells can promote virus clearance by producing many bioactive molecules such as perforin, granzyme and interferon, and therefore the decreased CD8^+^T cell level could reflect the severity of COVID-19 [13].

We found the decrease of CD4^+^T cell level, not CD8^+^T cell level, was an independent risk for in-hospital death in COVID-19 patients. We conducted the univariate analysis, the same as previous reports [9, 11]. The results confirmed that decreased CD4^+^T cell level and CD8^+^T cell level were associated with poor prognosis in COVID-19 patients. We also performed Cox proportional hazard regression, which showed that only decreased CD4^+^T cell level was an independent risk for in-hospital death in COVID-19 patients after adjusting for other confounding factors. Lymphocyte subsets play an important role in maintaining immune system function. CD8^+^T cells are essential for the direct attack and killing of virally infected cells, CD4^+^T cells can influence the differentiation and maturation of other cells by producing cytokines and chemokines, and the secretion of interferon-γ is a T-cytokine with both antiviral and immune activity [14, 15]. Patients infected with SARS-CoV-2 exhibit Th1 cells response and use cellular immunity to control the infection [16]. Viral infection causes comprehensive changes in cellular immunity, manifested by a decrease in lymphocytes, changes in the distribution of T cell subsets and an increase in cytokine concentrations [17]. But the mechanisms of SARS-CoV-2 infection leading to the decrease of lymphocytes and lymphocyte subsets remain unclear. Elevated concentrations of IL-10, IL-6, and TNF-α have been reported to be negatively correlated with total T-cell levels, CD4^+^T cell level, and CD8^+^T cell level, respectively. Compared with patients in the illness period, IL-10, IL-6, and TNF-α levels decreased significantly in patients in the decline stage, while total T-cell levels, CD4^+^T cell level, and CD8^+^T cell level were recovered [18, 19]. This phenomenon suggested the decrease of T-cells in COVID-19 patients may be due to the negative effects of high serum concentrations of TNF-α, IL-6, IL-10 on the survival or proliferation of T-cells [18]. Meanwhile, angiotensin-converting enzyme 2 (ACE2) had been reported to be expressed in white blood cells, and lymphopenia may be due to the direct lethal effect of SARS-COV-2 on lymphocytes by binding to the ACE2 receptors [20, 21].

Increased age and white blood cell count were associated with in-hospital death in our study, which were similar to several reports [22, 23]. The total case fatality rate of COVID-19 patients increased with age, which may be due to their frequently associated other chronic diseases, as well as lymphocytes and lymphocyte subsets decreased with age [22]. White blood cell count and neutrophil count of dead COVID-19 patients were higher than those of surviving COVID-19 patients, and leukocyte counts were negatively associated with the risk of death, which may be related to cytokine storm caused by the invasion of SARS-Cov-2 [23]. Patients with malignancy or immune system diseases may be at increased risk of severe COVID-19 and death [24]. To avoid these confounders, all patients with malignancy or immune system diseases were excluded from the study.

This study was limited by sample size and lacked a dynamic detection of CD4^+^T and CD8^+^T cell levels. Firstly, only 395 patients with COVID-19 were analyzed in our study, a relatively small sample size that may affect the statistical power. Secondly, the lack of dynamic measurement for CD4^+^T and CD8^+^T cell levels in the patients included in this study made the evaluation of the relationship between CD4^+^T cell level and disease changes in COVID-19 patients incomplete.

## Conclusions

In summary, the main findings of this study were that it was the CD8^+^T cell level, not the CD4^+^T cell level, reflected the severity of the disease, and that decreased CD4^+^T cell level were important in predicting the prognosis of COVID-19 patients. Both decreased CD4^+^T and CD8^+^T cell levels were associated with in-hospital death in COVID-19 patients, but only the reduction of CD4^+^T cell level was independently associated with increased in-hospital death in COVID-19 patients. Thus, in this acute-care setting, CD4^+^T cell level may provide early prognostic information in patients with COVID-19.

## Supplementary Information

**Additional file 1: Table S1.** Baseline characteristics of different degrees of CD4^+^T cell level in moderate COVID-19 patients. **Table S2.** Baseline characteristics of different degrees of CD4^+^T cell level in severe COVID-19 patients. **Table S3.** Effects of various variables on in-hospital death in Cox regression analysis in all patients with COVID-19. **Table S4.** Effects of various variables on in-hospital death in Cox regression analysis in severe COVID-19 patients. **Table S5.** Results of multivariate Cox proportional-hazards regression analyzing the effect of baseline variables on in-hospital death in severe COVID-19 patients.

**Additional file 2: Figure S1.** Forest plots of multivariate Cox proportional-hazards regression analyzing the effect of baseline variables on in-hospital death in severe COVID-19 patients.

## Data Availability

The datasets used and/or analyzed during the current study are available from the corresponding author on reasonable request.

## Abbreviations

SARS-CoV-2: Severe acute respiratory syndrome coronavirus 2
COVID-19: Coronavirus Disease 2019
RT-PCR: Reverse transcription polymerase chain reaction
SARS: Severe acute respiratory syndrome
MERS: Middle East respiratory syndrome
ACE2: Angiotensin-converting enzyme 2
CT: Computed tomography
COPD: Chronic obstructive pulmonary disease
CHD: Coronary heart disease
SBP: Systolic blood pressure
DBP: Diastolic blood pressure
WBC: White blood cell count
Hb: Hemoglobin
PLT: Platelet
LYM: Lymphocyte
ALT: Alanine aminotransferase
Cr: Creatinine
Hs-CRP: Hypersensitive C-reactive protein
PCT: Procalcitonin
