# Supplementary material for: Clinical characteristics and predictive value of lower CD4+T cell level in patients with moderate and severe COVID-19: a multicenter retrospective study
Source: BMC Infect Dis. 2021 Jan 12;21:57. doi: 10.1186/s12879-020-05741-w (PMC7803000; doi:10.1186/s12879-020-05741-w)
Supplement: Supplementary file 1 — Additional file 1: Table S1. Baseline characteristics of different degrees of CD4+T cell level in moderate COVID-19 patients. Table S2. Baseline characteristics of different degrees of CD4+T cell level in severe COVID-19 patients. Table S3. Effects of various variables on in-hospital death in Cox regression analysis in all patients with COVID-19. Table S4. Effects of various variables on in-hospital death in Cox regression analysis in severe COVID-19 patients. Table S5. Results of multivariate Cox proportional-hazards regression analyzing the effect of baseline variables on in-hospital death in severe COVID-19 patients. [file 12879_2020_5741_MOESM1_ESM.docx]

**Additional files**

**Supplementary table1**. Baseline characteristics of different degrees of CD4^+^T cell level in moderate COVID-19 patients.

| Variables | All(N=197) | CD4+T: lower group (N=95) | CD4+T: higher group (N=102) | *P* | Missing data |  |  |
| --- | --- | --- | --- | --- | --- | --- | --- |
| Baseline | | | | | | |  |
| Male/female(n) | 107/90 | 56/39 | 51/51 | 0.208 |  |  |  |
| Age(years) | 46.0 (34.5-53.0) | 47.0 (38.0-55.0) | 45.0 (34.0-52.0) | 0.190 |  |  |  |
| Temperature (℃) | 36.8 (36.5-37.1) | 36.8 (36.6-37.2) | 36.8 (36.5-37.1) | 0.720 |  |  |  |
| Heart rate (min) | 84.0 (76.0-92.5) | 85.0 (77.0-99.0) | 83.0 (75.0-90.0) | 0.048 |  |  |  |
| SBP (mmHg) | 124.0 (115.0-134.5) | 124.0 (115.0-136.0) | 124.0 (115.0-132.5) | 0.586 |  |  |  |
| DBP (mmHg) | 76.0 (70.5-86.0) | 76.0 (70.0-86.0) | 77.5 (70.8-85.0) | 0.871 |  |  |  |
| Symptoms and signs---No, % | | | | | | | |
| Fever on admission | 104 (52.8%) | 59 (62.1%) | 36 (37.9%) | 0.012 |  |  |  |
| Nasal congestion | 1 (0.5%) | 1 (1.1%) | 0 (0%) | 0.482 |  |  |  |
| Headache | 27 (13.7%) | 15 (15.8%) | 12 (11.8%) | 0.412 |  |  |  |
| Cough | 128 (65.0%) | 68 (71.6%) | 60 (58.8%) | 0.061 |  |  |  |
| Sore throat | 14 (7.1%) | 7 (7.4%) | 7 (6.9%) | 0.890 |  |  |  |
| Sputum production | 42 (21.3%) | 25 (26.3%) | 17 (16.7%) | 0.098 |  |  |  |
| Fatigue | 31 (15.7%) | 20 (21.1%) | 11 (10.8%) | 0.048 |  |  |  |
| Shortness of breath | 24 (12.2%) | 14 (14.7%) | 10 (9.8%) | 0.290 |  |  |  |
| Nausea or vomiting | 14 (7.1%) | 8 (8.4%) | 6 (5.9%) | 0.488 |  |  |  |
| Myalgia or arthralgia | 18 (9.1%) | 10 (10.5%) | 8 (7.8%) | 0.514 |  |  |  |
| Chill | 2 (1.0%) | 0 (0%) | 2 (2.0%) | 0.498 |  |  |  |
| Throat congestion | 0 (0%) | 0 (0%) | 0 (0%) | --- |  |  |  |
| Coexisting disorders---No, % | | | | | | | |
| Diabetes | 8 (4.1%) | 2 (2.1%) | 6 (5.9%) | 0.281 |  |  |  |
| Hypertension | 18 (9.1%) | 10 (10.5%) | 8 (7.8%) | 0.514 |  |  |  |
| Coronary heart disease | 2 (1.0%) | 2 (2.1%) | 0 (0%) | 0.231 |  |  |  |
| Hepatitis B infection | 5 (2.5%) | 2 (2.1%) | 3 (2.9%) | 1.000 |  |  |  |
| COPD | 0 (0%) | 0 (0%) | 0 (0%) | --- |  |  |  |
| Laboratory findings | | | | | | | |
| WBC (×10^9^/L) | 5.2 (4.1-6.8) | 4.8 (3.7-5.9) | 5.9 (4.6-7.3) | <0.001 |  |  |  |
| Hb (g/L) | 135.0±17.5 | 135.4±19.1 | 134.7±16.0 | 0.710 |  |  |  |
| PLT (×10^9^/L) | 178.0 (145.0-234.5) | 160.0 (129.0-204.0) | 204.0 (155.0-255.3) | <0.001 |  |  |  |
| LYM (×10^9^/L) | 1.2 (0.9-1.6) | 0.9 (0.7-1.1) | 1.6 (1.3-1.9) | <0.001 |  |  |  |
| LYM<1.1×10^9^/L | 85 (43.1) | 76 (80.0%) | 9 (8.8%) | <0.001 |  |  |  |
| ALT (U/L) | 20.0 (13.8-36.6) | 21.4 (14.8-38.2) | 18.0 (13.4-36.3) | 0.221 |  |  |  |
| Cr (umol/L) | 66.0 (55.0-78.0) | 66.0 (55.0-77.0) | 66.5 (53.0-78.3) | 0.998 |  |  |  |
| D-dimer (mg/L) | 0.3 (0.2-0.5) | 0.3 (0.2-0.6) | 0.3 (0.2-0.5) | 0.023 |  |  |  |
| K^+^ (mmol/L) | 4.0±0.2 | 4.0±0.4 | 4.1±0.4 | 0.617 |  |  |  |
| Hs-CRP (mg/L) | 7.0 (1.7-22.8) | 10.0 (4.3-35.4) | 2.7 (0.7-16.9) | <0.001 | 1 (0.5%) |  |  |
| PCT (ng/ml) | 0.04 (0.03-0.06) | 0.04 (0.03-0.06) | 0.04 (0.02-0.05) | 0.004 | 4 (2.0%) |  |  |
| CD4 cell level | 411.0 (279.0-581.0) | 277.0 (214.0-338.0) | 568.0 (476.3-739.0) | <0.001 |  |  |  |
| CD8 cell level | 267.0 (182.5-364.0) | 186.0 (135.0-267.0) | 332.0 (257.8-482.5) | <0.001 |  |  |  |
| CD4/CD8 ratio | 1.5 (1.2-2.0) | 1.4 (1.1-1.8) | 1.7 (1.4-2.2) | <0.001 |  |  |  |
| Abnormalities on chest CT---No, % | | | | | | | |
| Ground-glass opacity | 87 (44.2%) | 41 (43.2%) | 46 (45.1%) | 0.784 |  |  |  |
| Local patchy shadowing | 78 (39.6%) | 45 (47.4%) | 33 (32.4%) | 0.031 |  |  |  |
| Treatment | | | | | | | |
| Oxygen inhalation | 155 (78.7%) | 81 (85.3%) | 74 (72.5%) | 0.029 |  |  |  |
| Glucocorticoids | 15 (7.6%) | 11 (11.6%) | 4 (3.9%) | 0.043 |  |  |  |
| Antiviral treatment | 197 (100.0%) | 95 (100.0%) | 102 (100.0%) | --- |  |  |  |
| Intravenous immunoglobulin | 3 (1.5%) | 2 (2.1%) | 1 (1.0%) | 0.610 |  |  |  |
| Antibiotic treatment | 36 (18.3%) | 27 (28.4%) | 9 (8.8%) | <0.001 |  |  |  |
| Antifungal treatment | 0 (0%) | 0 (0%) | 0 (0%) | --- |  |  |  |
| Clinical outcome | | | | | | | |
| Death (No, %) | 0 (0%) | 0 (0%) | 0 (0%) | --- |  |  |  |

Abbreviations: SBP, systolic blood pressure; DBP, diastolic blood pressure; COPD, Chronic obstructive pulmonary disease; WBC, white blood cell; Hb, Hemoglobin; PLT, platelet; LYM, lymphocyte; ALT, alanine aminotransferase; Cr, creatinine; Hs-CRP, hypersensitive C-reactive protein; PCT, procalcitonin.

**Supplementary table2.** Baseline characteristics of different degrees of CD4^+^T cell level in severe COVID-19 patients.

| Variables | All(N=198) | CD4+T: lower group (N=100) | CD4+T: higher group (N=98) | *P* | Missing data |  |  |
| --- | --- | --- | --- | --- | --- | --- | --- |
| Baseline | | | | | | |  |
| Male/female(n) | 97/101 | 59/41 | 38/60 | 0.004 |  |  |  |
| Age(years) | 63.0 (52.0-72.0) | 64.0 (52.5-76.0) | 62.0 (51.0-67.0) | 0.041 |  |  |  |
| Smoking history---No, % | 11 (5.6%) | 6 (6.0%) | 5 (5.1%) | 0.783 |  |  |  |
| Temperature (℃) | 36.9 (36.5-37.6) | 37.0 (36.6-37.9) | 36.8 (36.5-37.3) | 0.017 | 31 (15.7%) |  |  |
| Heart rate (min) | 85.5 (78.0-95.0) | 86.0 (78.0-94.5) | 85.0 (77.3-95.0) | 0.784 | 4 (2.0%) |  |  |
| SBP (mmHg) | 129.0 (118.0-138.0) | 127.5 (115.0-137.3) | 130.0 (119.8-139.0) | 0.160 | 6 (3.0%) |  |  |
| DBP (mmHg) | 78.0 (70.0-84.8) | 77.0 (70.0-84.3) | 79.0 (71.5-85.0) | 0.548 | 6 (3.0%) |  |  |
| Symptoms and signs---No, % | | | | | | | |
| Fever on admission | 159 (80.3%) | 82 (82.0%) | 77 (78.6%) | 0.544 |  |  |  |
| Nasal congestion | 1 (0.5%) | 1 (1.0%) | 0 (0%) | 1.000 |  |  |  |
| Headache | 9 (4.5%) | 5 (5.0%) | 4 (4.1%) | 1.000 |  |  |  |
| Cough | 129 (65.2%) | 70 (70.0%) | 59 (60.2%) | 0.148 |  |  |  |
| Sore throat | 8 (4.0%) | 3 (3.0%) | 5 (5.1%) | 0.495 |  |  |  |
| Sputum production | 60 (30.5%) | 31 (31.3%) | 29 (29.6%) | 0.793 | 1 (0.5%) |  |  |
| Fatigue | 76 (38.6%) | 39 (39.4%) | 37 (37.8%) | 0.813 | 1 (0.5%) |  |  |
| Shortness of breath | 94 (47.5%) | 61 (61.0%) | 33 (33.7%) | <0.001 |  |  |  |
| Nausea or vomiting | 22 (11.1%) | 15 (15.0%) | 7 (7.1%) | 0.079 |  |  |  |
| Myalgia or arthralgia | 16 (8.1%) | 10 (10.0%) | 6 (6.1%) | 0.317 |  |  |  |
| Chill | 10 (5.1%) | 8 (8.0%) | 2 (2.0%) | 0.101 |  |  |  |
| Throat congestion | 3 (1.5%) | 0 (0%) | 3 (3.1%) | 0.119 |  |  |  |
| Coexisting disorders---No, % | | | | | | | |
| Diabetes | 39 (19.7%) | 20 (20.0%) | 19 (19.4%) | 0.943 |  |  |  |
| Hypertension | 84 (42.4%) | 38 (38.0%) | 46 (46.9%) | 0.203 |  |  |  |
| Coronary heart disease | 23 (11.8%) | 13 (13.1%) | 10 (10.4%) | 0.557 | 3 (1.5%) |  |  |
| Hepatitis B infection | 4 (2.1%) | 4 (4.0%) | 0 (0%) | 0.121 | 3 (1.5%) |  |  |
| COPD | 6 (3.1%) | 5 (5.1%) | 1 (1.0%) | 0.212 | 3 (1.5%) |  |  |
| Laboratory findings | | | | | | | |
| WBC (×10^9^/L) | 5.5 (4.2-7.0) | 5.5 (3.9-7.6) | 5.3 (4.4-6.8) | 0.881 | 2 (1.0%) |  |  |
| Hb (g/L) | 124.5 (115.0-137.0) | 126.5 (113.0-139.0) | 124.0 (116.3-136.0) | 0.945 | 2 (1.0%) |  |  |
| PLT (×10^9^/L) | 197.0 (146.3-264.0) | 161.5 (127.5-226.5) | 241.0 (181.5-299.8) | <0.001 | 2 (1.0%) |  |  |
| LYM (×10^9^/L) | 1.0 (0.7-1.4) | 0.7 (0.5-0.9) | 1.4 (1.2-1.4) | <0.001 | 6 (3.0%) |  |  |
| LYM <1.1×10^9^/L | 105 (54.7%) | 87 (88.8%) | 18 (19.1%) | <0.001 | 6 (3.0%) |  |  |
| ALT (U/L) | 26.0 (17.0-40.0) | 18.0 (11.0-26.0) | 16.5 (11.0-25.0) | 0.905 | 4 (2.0%) |  |  |
| Cr (umol/L) | 61.0 (50.0-77.5) | 67.0 (56.0-82.0) | 56.0 (48.3-68.8) | <0.001 | 5 (2.5%) |  |  |
| D-dimer (mg/L) | 0.71 (0.36-3.26) | 0.76 (0.44-2.48) | 0.66 (0.32-3.37) | 0.320 | 14 (7.1%) |  |  |
| K (mmol/L) | 4.0 (3.6-4.4) | 4.0 (3.5-4.4) | 4.0 (3.7-4.4) | 0.260 | 6 (3.0%) |  |  |
| Hs-CRP (mg/L) | 5.0 (5.0-23.8) | 5.0 (5.0-61.4) | 5.0 (1.6-5.0) | <0.001 | 44 (22.2%) |  |  |
| PCT (ng/ml) | 0.06 (0.03-0.125) | 0.09 (0.05-0.24) | 0.04 (0.03-0.07) | <0.001 | 17 (8.6%) |  |  |
| CD4^+^T cell level | 400.0 (227.3-563.3) | 232.0 (150.3-308.3) | 563.5 (481.5-689.8) | <0.001 |  |  |  |
| CD8^+^T cell level | 211.5 (122.0-333.3) | 147.0 (84.0-208.3) | 312.5 (226.8-417.0) | <0.001 |  |  |  |
| CD4/CD8 ratio | 1.8 (1.2-2.4) | 1.6 (1.1-2.1) | 1.9 (1.5-2.9) | <0.001 |  |  |  |
| Abnormalities on chest CT---No, % | | | | | | | |
| Ground-glass opacity | 83 (54.6%) | 36 (50.0%) | 47 (58.8%) | 0.279 | 46 (23.2%) |  |  |
| Local patchy shadowing | 57 (37.5%) | 24 (33.3%) | 33 (41.3%) | 0.314 | 46 (23.2%) |  |  |
| Treatment | | | | | | | |
| Oxygen inhalation | 173 (90.1%) | 93 (94.9%) | 80 (85.1%) | 0.023 | 6 (3.0%) |  |  |
| Mechanical ventilation | 28 (14.7%) | 24 (24.5%) | 4 (4.3%) | <0.001 | 7 (3.5%) |  |  |
| Glucocorticoids | 79 (39.9%) | 53 (53.0%) | 26 (26.5%) | <0.001 |  |  |  |
| Antiviral treatment | 191 (96.5%) | 96 (96.0%) | 95 (96.9%) | 1.000 |  |  |  |
| Intravenous immunoglobulin | 68 (35.2%) | 35 (35.7%) | 33 (34.7%) | 0.887 | 5 (2.5%) |  |  |
| Antibiotic treatment | 143 (72.2%) | 85 (85.0%) | 58 (59.2%) | <0.001 |  |  |  |
| Antifungal treatment | 4 (2.0%) | 2 (2.0%) | 2 (2.0%) | 1.000 |  |  |  |
| Clinical outcome | | | | | | | |
| Death (No, %) | 27 (13.6%) | 25 (25.0%) | 2 (2.0%) | <0.001 |  |  |  |

Abbreviations: SBP, systolic blood pressure; DBP, diastolic blood pressure; COPD, Chronic obstructive pulmonary disease; WBC, white blood cell; Hb, Hemoglobin; PLT, platelet; LYM, lymphocyte; ALT, alanine aminotransferase; Cr, creatinine; Hs-CRP, hypersensitive C-reactive protein; PCT, procalcitonin.

**Supplementary table3.** Effects of various variables on in-hospital death in Cox regression analysis in all patients with COVID-19.

| Characteristics | HR (95%CI) | *P* |
| --- | --- | --- |
| Sex, Male vs. Female | 1.625 (0.744-3.549) | 0.223 |
| Age, per 1 years | 1.096 (1.062-1.131) | <0.001 |
| Temperature, per 1℃ | 1.115 (0.655-1.899) | 0.687 |
| Heart rate (min), per 1 beat | 1.009 (0.981-1.038) | 0.523 |
| SBP (mmHg), per 1 mmHg | 1.013 (0.994-1.033) | 0.177 |
| DBP (mmHg), per 1 mmHg | 1.008 (0.975-1.042) | 0.634 |
| History of hypertension, Yes vs. No | 5.371 (2.459-11.732) | <0.001 |
| History of diabetes, Yes vs. No | 2.134 (0.861-5.287) | 0.102 |
| History of CHD, Yes vs. No | 2.641 (0.913-7.637) | 0.073 |
| History of COPD, Yes vs. No | 5.105 (1.209-21.558) | 0.027 |
| History of HBI, Yes vs. No | 1.616 (0.219-11.906) | 0.638 |
| WBC (×10^9^/L), per 1×10^9^/L | 1.240 (1.157-1.329) | <0.001 |
| Hb (g/L), per 1 g/L | 0.992 (0.975-1.010) | 0.407 |
| PLT (×10^9^/L), per 1×10^9^/L | 0.995 (0.990-1.000) | 0.059 |
| LYM (×10^9^/L), per 1×10^9^/L | 0.035 (0.010-0.120) | <0.001 |
| LYM<×10^9^/L, yes vs. no | 6.332 (2.190-18.310) | 0.001 |
| ALT (U/L), per 1 U/L | 1.005 (0.991-1.018) | 0.486 |
| Cr (umol/L), per 1 umol/L | 1.004 (0.999-1.009) | 0.094 |
| D-dimer (mg/L), per 1 mg/L | 1.033 (1.024-1.042) | <0.001 |
| K^+^ (mmol/L), per 1 mmol/L | 1.310 (0.685-2.502) | 0.414 |
| Hs-CRP (mg/L), per 1 mg/L | 0.994 (0.980-1.008) | 0.378 |
| PCT (ng/ml), per 1 ng/ml | 1.010 (0.935-1.091) | 0.797 |
| CD4^+^T cell level, lower vs. higher | 13.659 (3.235-57.671) | <0.001 |
| CD8^+^T cell level, lower vs. higher | 10.883 (3.277-36.145) | <0.001 |
| CD4/CD8 ratio, per 1 unit | 1.240 (1.026-1.497) | 0.026 |
| Ground-glass opacity, Yes vs. No | 0.585 (0.196-1.746) | 0.337 |
| Local patchy shadowing, Yes vs. No | 0.426 (0.119-1.528) | 0.190 |
| Oxygen inhalation, Yes vs. No | 26.390 (0.313-2224.440) | 0.148 |
| mechanical ventilation, Yes vs. No | 14.553 (6.725-31.492) | <0.001 |
| Glucocorticoids, Yes vs. No | 10.215 (4.318-24.166) | <0.001 |
| Antiviral treatment, Yes vs. No | 0.428 (0.058-3.152) | 0.405 |
| Intravenous immunoglobulin, Yes vs. No | 10.068 (4.521-22.421) | <0.001 |
| Antibiotic treatment, Yes vs. No | 87.911 (3.794-2037.206) | 0.005 |
| Antifungal treatment, Yes vs. No | 10.575 (2.499-44.755) | 0.001 |

Abbreviations: SBP, systolic blood pressure; DBP, diastolic blood pressure; COPD, Chronic obstructive pulmonary disease; WBC, white blood cell; Hb, Hemoglobin; PLT, platelet; LYM, lymphocyte; ALT, alanine aminotransferase; Cr, creatinine; Hs-CRP, hypersensitive C-reactive protein; PCT, procalcitonin.

**Supplementary table4.** Effects of various variables on in-hospital death in Cox regression analysis in severe COVID-19 patients.

| Characteristics | HR (95%CI) | *P* |
| --- | --- | --- |
| Sex, Male vs. Female | 1.863 (0.853-4.069) | 0.118 |
| Age, per 1 years | 1.070 (1.034-1.107) | <0.001 |
| Temperature, per 1℃ | 0.929 (0.557-1.550) | 0.778 |
| Heart rate (min), per 1 beat | 1.007 (0.978-1.037) | 0.643 |
| SBP (mmHg), per 1 mmHg | 1.006 (0.987-1.025) | 0.553 |
| DBP (mmHg), per 1 mmHg | 1.016 (0.981-1.052) | 0.379 |
| History of hypertension, Yes vs. No | 2.558 (1.171-5.588) | 0.018 |
| History of diabetes, Yes vs. No | 1.152 (0.465-2.854) | 0.760 |
| History of CHD, Yes vs. No | 1.304 (0.451-3.771) | 0.624 |
| History of COPD, Yes vs. No | 2.408 (0.570-10.171) | 0.232 |
| History of HBI, Yes vs. No | 1.799 (0.244-13.260) | 0.565 |
| WBC (×10^9^/L), per 1×10^9^/L | 1.209 (1.130-1.293) | <0.001 |
| Hb (g/L), per 1 g/L | 1.006 (0.987-1.025) | 0.543 |
| PLT (×10^9^/L), per 1×10^9^/L | 0.994 (0.989-0.999) | 0.017 |
| LYM (×10^9^/L), per 1×10^9^/L | 0.078 (0.026-0.237) | <0.001 |
| LYM<1.1×10^9^/L, yes vs. no | 5.213 (1.802-15.077) | 0.002 |
| ALT (U/L), per 1 U/L | 1.000 (0.987-1.014) | 0.948 |
| Cr (umol/L), per 1 umol/L | 1.003 (0.999-1.008) | 0.130 |
| D-dimer (mg/L), per 1 mg/L | 1.027 (1.018-1.037) | <0.001 |
| K^+^ (mmol/L), per 1 mmol/L | 1.251 (0.724-2.160) | 0.422 |
| Hs-CRP (mg/L), per 1 mg/L | 0.992 (0.980-1.004) | 0.194 |
| PCT (ng/ml), per 1 ng/ml | 0.993 (0.912-1.082) | 0.876 |
| CD4^+^T cell level, lower vs. higher | 13.962 (3.306-58.963) | <0.001 |
| CD8^+^T cell level, lower vs. higher | 8.305 (2.500-27.587) | 0.001 |
| Ground-glass opacity, Yes vs. No | 0.621 (0.216-1.791) | 0.378 |
| Local patchy shadowing, Yes vs. No | 0.262 (0.059-1.172) | 0.080 |
| Oxygen inhalation, Yes vs. No | 23.855 (0.111-5107.526) | 0.247 |
| Mechanical ventilation, Yes vs. No | 7.064 (3.263-15.290) | <0.001 |
| Glucocorticoids, Yes vs. No | 4.858 (2.053-11.492) | <0.001 |
| Antiviral treatment, Yes vs. No | 0.899 (0.122-6.628) | 0.917 |
| Intravenous immunoglobulin, Yes vs. No | 4.047 (1.817-9.011) | 0.001 |
| Antibiotic treatment, Yes vs. No | 35.221 (1.088-1139.768) | 0.045 |
| Antifungal treatment, Yes vs. No | 5.093 (1.204-21.548) | 0.027 |

Abbreviations: SBP, systolic blood pressure; DBP, diastolic blood pressure; COPD, Chronic obstructive pulmonary disease; WBC, white blood cell; Hb, Hemoglobin; PLT, platelet; LYM, lymphocyte; ALT, alanine aminotransferase; Cr, creatinine; Hs-CRP, hypersensitive C-reactive protein; PCT, procalcitonin.

**Supplementary table5.** Results of multivariate Cox proportional-hazards regression analyzing the effect of baseline variables on in-hospital death in severe COVID-19 patients.

| Mode | HR (95%CI) | *P* |
| --- | --- | --- |
| Not Adjusted CD4^+^T level, lower vs. higher | 13.962 (3.306-58.963) | <0.001 |
| Mode 1 | | |
| CD4^+^T cell level, lower vs. higher | 15.416 (2.036-116.722) | 0.008 |
| Sex, male vs. female | 1.391 (0.563-3.436) | 0.475 |
| Age, per 1 year | 1.062 (1.020-1.105) | 0.004 |
| Temperature, per 1℃ | 0.710 (0.403-1.249) | 0.234 |
| Mode 2 | | |
| CD4^+^T cell level, lower vs. higher | 15.136 (3.520-65.079) | <0.001 |
| Hypertension, yes vs. no | 4.541 (2.011-10.256) | <0.001 |
| Diabetes, yes vs. no | 0.648 (0.254-1.653) | 0.364 |
| Shortness of breath, yes vs. no | 4.593 (1.708-12.346) | 0.003 |
| Mode 3 |  |  |
| CD4^+^T cell level, lower vs. higher | 7.366 (1.665-32.584) | 0.008 |
| WBC, per 1×10^9^/L | 1.220 (1.123-1.326) | <0.001 |
| PLT, per 1×10^9^/L | 0.993 (0.988-0.998) | 0.013 |
| Cr, per 1 umol/L | 1.000 (0.993-1.008) | 0.923 |
| Mode 4 | | |
| CD4^+^T cell level, lower vs. higher | 12.116 (2.770-52.991) | 0.001 |
| Hs-CRP, per 1 mg/L | 0.987 (0.973-1.001) | 0.062 |
| PCT, per 1 ng/ml | 1.001 (0.899-1.115) | 0.985 |
| D-dimer, per 1 mg/L | 1.020 (1.009-1.030) | <0.001 |
| Mode 5 |  |  |
| CD4^+^T cell level, lower vs. higher | 13.075 (1.996-85.669) | 0.007 |
| CD8^+^T cell level, lower vs. higher | 2.873 (0.771-10.709) | 0.116 |
| CD4/CD8 ratio, per 1 unit | 1.181 (0.988-1.412) | 0.067 |
| LYM level, lower vs. higher | 0.839 (0.244-2.890) | 0.781 |
| Mode 6 |  |  |
| CD4^+^T cell level, lower vs. higher | 6.964 (1.554-31.210) | 0.011 |
| Age, per 1 year | 1.056 (1.016-1.098) | 0.006 |
| Hypertension, yes vs. no | 1.615 (0.607-4.293) | 0.337 |
| Shortness of breath, yes vs. no | 2.029 (0.676-6.092) | 0.207 |
| WBC, per 1×10^9^/L | 1.221 (1.094-1.362) | <0.001 |
| PLT, per 1×10^9^/L | 0.996 (0.991-1.001) | 0.149 |
| D-dimer, per 1 mg/L | 0.997 (0.981-1.014) | 0.755 |

Abbreviations: SBP, systolic blood pressure; DBP, diastolic blood pressure; COPD, Chronic obstructive pulmonary disease; WBC, white blood cell; Hb, Hemoglobin; PLT, platelet; LYM, lymphocyte; ALT, alanine aminotransferase; Cr, creatinine; Hs-CRP, hypersensitive C-reactive protein; PCT, procalcitonin.
