# Supplementary material for: Clinical characteristics and predictive value of lower CD4+T cell level in patients with moderate and severe COVID-19: a multicenter retrospective study
Source: BMC Infect Dis. 2021 Jan 12;21:57. doi: 10.1186/s12879-020-05741-w (PMC7803000; doi:10.1186/s12879-020-05741-w)
Supplement: Supplementary file 2 — Additional file 2: Figure S1. Forest plots of multivariate Cox proportional-hazards regression analyzing the effect of baseline variables on in-hospital death in severe COVID-19 patients. [file 12879_2020_5741_MOESM2_ESM.docx]

**Supplementary figure 1.** Forest plots of multivariate Cox proportional-hazards regression analyzing the effect of baseline variables on in-hospital death in severe COVID-19 patients.


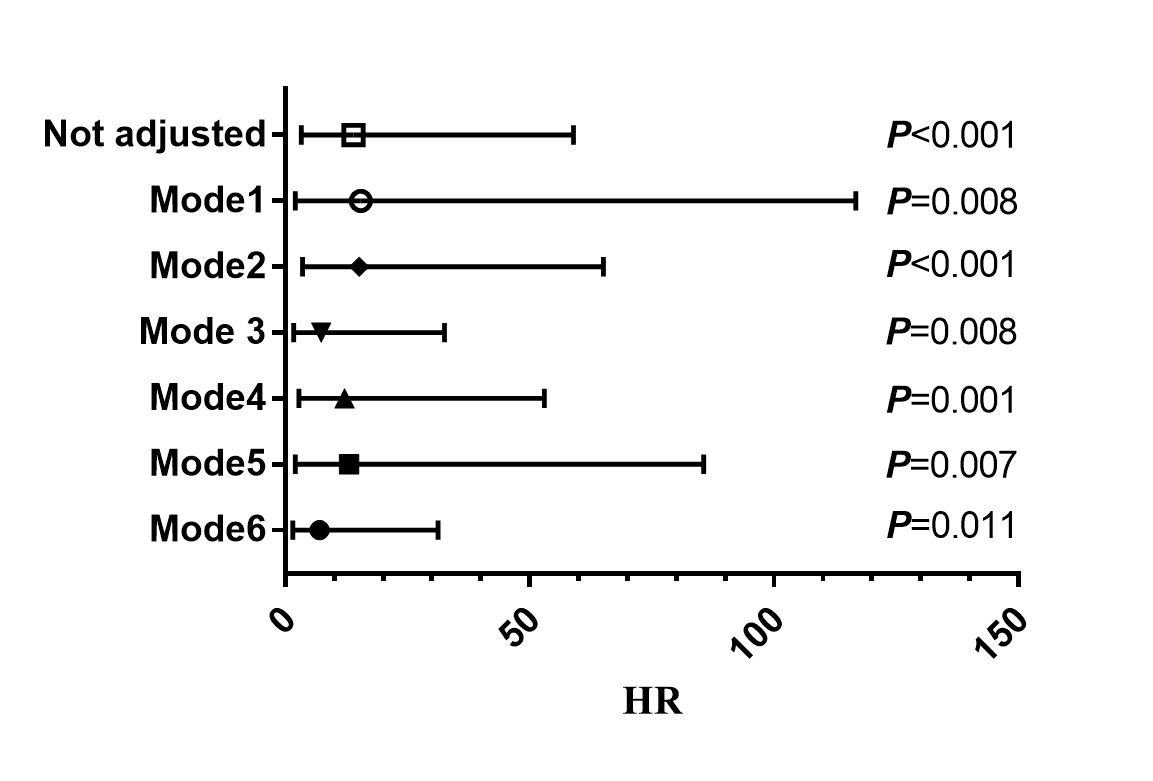
Mode1: adjusted sex, age, and temperature; Mode2: adjusted hypertension, diabetes, and shortness of breath; Mode3: adjusted WBC, PLT, and Cr; Mode4: adjusted Hs-CRP, PCT and D-dimer; Mode5: adjusted the group with lower CD8^+^T cell level, CD4/CD8 ratio, and the group with lower LYM level; Mode6: adjusted age, hypertension, shortness of breath, WBC, PLT, and D-dimer.
